# Use of antenatal care services in Kassala, eastern Sudan

**DOI:** 10.1186/1471-2393-10-67

**Published:** 2010-10-25

**Authors:** Abdel Aziem A Ali, Mohammed M Osman, Ameer O Abbaker, Ishag Adam

**Affiliations:** 1 Faculty of Medicine, Kassala University, Kassala, Sudan; 2 Faculty of Medicine, University of Khartoum, Khartoum, Sudan

## Abstract

**Background:**

Antenatal care is named as one of the four pillars initiatives of the Safe Motherhood Initiative. While many of routine antenatal care procedure have little effect on maternal mortality and morbidity, some of these have been ascertained as beneficial. The aim of this study was to investigate coverage of antenatal care and identify factors associated with inadequacy of antenatal care in Kassala, eastern Sudan.

**Methods:**

A cross-sectional community-based study was carried out in Kassala, eastern Sudan during September-October 2009. Household surveys were conducted. Structured questionnaires were used to gather data from women who had been pregnant within the last year, or pregnant more than 14 weeks.

**Results:**

Out of 900 women investigated for antenatal care coverage, 811(90%) women had at least one visit. Only 11% of the investigated women had ≥ four antenatal visits, while 10.0% had not attended at all. Out of 811 women who attended at least one visit, 483 (59.6%), 303 (37.4%) and 25 (3.1%) women attended antenatal care in the first, second and third trimester, respectively. In logistic regression analyses, while maternal age and residence were not associated with inadequacy of antenatal care (<2 visits), high parity (OR = 2.0, CI = 1.1-3.5; *P *= 0.01) and husband education ≤ secondary level (OR = 2.4, CI = 1.3-4.2; *P *= 0.002) were associated with inadequacy of antenatal care.

**Conclusions:**

Antenatal care showed a low coverage in Kassala, eastern Sudan. This low coverage was associated with high parity and low husband education.

## Background

Antenatal care is one of the four pillars initiatives of the Safe Motherhood Initiative; however, its relative contribution to maternal health has been under debate. While many of routine antenatal care procedures have little effect on maternal mortality and morbidity, some of these have been ascertained as beneficial [1,2]. Antenatal care provides advice, reassurance, education, support for the woman on screening programs and detects the problems that make the pregnancy high risk one [3]. There are many socio-economic and cultural factors which act as barriers to the use of antenatal care [4]. Although, it can't be claimed that antenatal care is the only solution for the high maternal and perinatal death in the developing world, but it can help to reach the Millennium Development Goals for the maternal and child mortality [5].

Although, World Health Organization recommended four antenatal visits for the low risk pregnancy [6], there is still debate regarding the optimal number of visits for the antenatal care [7]. There is tendency towards late attendance for the first antenatal care visit in developing countries [8] and the coverage in Sub-Saharan Africa lags far behind [9].

Sudan, which is the largest country in Africa with 40 million inhabitants, has one of the highest rates of maternal and perinatal mortality [10]. We have previously suggested that the high maternal and perinatal mortality in Sudan could be reduced by increasing the use of antenatal care [11]. In spite of this, there is little published data concerning the use of antenatal care in Sudan [12,13]. Investigating coverage of antenatal care and its barriers is fundamental to provide caregivers and health planners with basic data necessary for the intervention measures. Thus the current study was conducted in Kassala, eastern Sudan to investigate the antenatal care coverage and its barriers.

## Methods

A community-based cross sectional household survey was conducted to investigate coverage and factors associated with the use of antenatal services in Kassala, eastern Sudan between September and October 2009. Kassala is 550 kg from Khartoum on Ethiopian-Eritrean border with 260000 inhabitants. In Kassala, there are 28 health centers and three hospitals providing antenatal care free of charge. A random sample of 900 women was selected to calculate the proportion of those using antenatal in the area within 3 percentage points of the true proportion, assuming the true proportion was 70% and that 10% of women would not be respond.

Semi-structured questionnaires were used to gather data from women who had been pregnant within the last year, or were more than 14 weeks (calculated from the last menstrual period) pregnant to identify the antenatal experiences of women and associated factors of age, parity and education. Trained interviewers (medical officers) visited the selected women at home and administered the questionnaires in the local Arabic language with full privacy. After taking an informed consent, participants were asked orally if they had visited an ANC during their current or last pregnancy. If they answered "No," they were asked to specify their reasons for this.

Data were entered into a computer database and SPSS software (SPSS Inc., Chicago, IL, USA, version 13.0) and double checked before analysis. Means and proportions for the socio-demographic characteristics were compared between the groups of the study using ANOVA and x^2 ^test, respectively. Univariate and multivariate analyses were performed. Antenatal care inadequacy (<2 visits) was the dependent variable and socio-demographic characteristics were independent variables. Confidence intervals of 95% were calculated and *P *< 0.05 was considered significant. In case of discrepancy between the results of ANOVA and x^2 ^test and the results of multivariate analyses, the later was taken as final.

### Ethics

The study received ethical clearance from the Research Board at the Faculty of Medicine, University of Khartoum.

## Results

During the survey 980 women were requested to participate, of which 80 women were no-responders. Out of 900 women who responded were investigated for antenatal care coverage in Kassala, 811(90%) women had at least one visit. However, only 11% of the investigated women had ≥ four antenatal visits, while 10.0% had not attended at all, table 1. Five hundred and thirty two (59.0%) of these 900 women were pregnant at mean ± SD gestational age of 26.3 ± 6.2 weeks, 337 and 195 women in the second and third trimester, respectively.

**Table 1:** Socio-demographic characteristics and use of antenatal care in Kassala, eastern Sudan*

Variable	No antenatal (*N *= 89)	One visit (*N *= 411)	2-3 visits (*N *= 302)	≥4 visits (*N *= 98)	*P*
Age	29.0 ± 5.6	28.1 ± 9.5	28.4 ± 6.3	27.1 ± 6.3	0.03
Parity	4.5 ± 3.2	3.8 ± 1.9	3.8 ± 2.2	3.5 ± 2.1	0.008
Residence					
Rural	37(41.6)	167(40.6)	47(15.6)	49(50)	<0.001
Urban	52(58.4)	244(59.4)	255(84.4)	49(50)	<0.001
Lady education					
≤ secondary level	82(92.1)	283(68.9)	95(31.4)	66(67.3)	<0.001
>secondary level	7(7.9)	128(31.1)	207(68.6)	32(32.7)	<0.001
Husband education					
≤secondary level	76(85.4)	264(64.2)	88(29.1)	60(61.2)	<0.001
>secondary level	13(14.6)	147(35.8)	214(70.9)	38(38.8)	<0.001

Data are shown as mean ± SD or number (percentage) as applicable.

Out of 811 women who attended at least one visit, 483 (59.6%), 303 (37.4%) and 25 (3.1%) women attended antenatal care in the first, second and third trimester, respectively. Out of 811, 94 (11.6%), 326(40.2%), 392 (48.3%) women attended in the hospital, health centers and private consultant clinics, respectively.

Women (89) who had no antenatal care claimed that; it is of no value 12 (13.5%), expensive 34 (38.2%), far or not available 11 (12.4%), their pregnancy was quite normal 32 (35.6%).

Age and parity were significantly higher in women who did not attended antenatal care. Likewise, women who did not use antenatal care services were those who had less education and those of rural residency, table 1.

In logistic regression model, while age, residence were not associated with antenatal care inadequacy, high parity (OR = 2.0, CI = 1.1-3.5; *P *= 0.01) and husband education ≤ secondary level (OR = 2.4, CI = 1.3-4.2; *P *= 0.002) were associated with inadequacy of antenatal care, table 2.

**Table 2:** Factors associated with inadequacy of antenatal care use in Kassala, Sudan using univariate and multivariate analyses

Variable	Univariate analyses			Multivariate analyses		
	OR	95% CI	*P*-value	OR	95% CI	*P*-value
Age, years	1.0	0.9-1.0	0.7	0.9	0.9-1.0	0.2
Parity ≥3	1.9	1.2-3.0	0.005	2.0	1.1-3.5	0.01
Women's education ≤ secondary level	2.4	1.5-4.0	<0.001	1.3	0.7-2.4	0.2
Husband's education ≤ secondary level	3.1	2.0-5.0	<0.001	2.4	1.3-4.2	0.002
Rural residence	1.9	0.9-2.3	0.06	1.2	0.8-2.0	0.2

Abbreviations: OR, Odds Ratio; CI, confidence interval

## Discussion

The current study showed a low coverage of antenatal care in this region of Sudan, where only 11% of the investigated women had ≥ four antenatal visits, while 10.0% had not attended at all. We recently reported low antenatal care coverage in Darfur Sudan, where 41.8% of the 402 surveyed women had not attended antennal care at all [13].

While in Khartoum hospital, we observed that 17.0% out of 2076 parturient women had not attended antenatal care clinic during the index pregnancy [14]. In neighboring Ethiopia, a higher (76.7%) level of antenatal care use has been reported [4]. However, comparison of the percentages of inadequate utilization of antenatal care among different studies is impaired by the different forms of classification employed. While some investigators considered antenatal care to be adequate when a pregnant woman attended six or more visits, started them during the first trimester, was submitted to laboratory tests and to at least one breast examination and one ultrasound, and had her blood pressure determined at least once [15]. World Health organization considered antenatal care as adequate when there are at least four visits for low risk pregnancy [6]. Thus, it is necessary to strive for the standardization of the measurement of adequate utilization of antenatal care that will facilitate comparison of different studies. Until the same criteria are used, data from within one country (Sudan) and between Sudan and the rest of the world cannot be adequately compared. Thus we adopted definition of inadequacy of antenatal care as less than two visits in this study to mimic to what has been adopted by the federal ministry of health in Sudan [16].

In the current study, maternal age was not associated with antenatal care inadequacy. This goes with our recent observation in Darfur Sudan [13]. The effect of mothers' age on use of antenatal care services is unclear. Some studies suggested that women in their thirties are more likely to have antenatal care than older women and teenagers [17]. This is may be because of the confounding effect of parity, women of higher parity and teenagers are not expecting to become pregnant, tending to use less antenatal care in developing countries. Low utilization of antenatal care among high parity women in this study as well as in the other studies [17] could be because of time management, limited resources in the family and negative perceptions resulting from previous pregnancies. It is possible that multiparous mothers, who have greater experience, feel more confident during pregnancy and consider antenatal care less important.

In the current study, while maternal education was associated with antenatal care inadequacy in univariate analyses only, husband education was associated with antenatal care in univariate as well as in multivariate analyses. Recently we observed that, couple education was associated with utilization of antenatal care in Darfur, Sudan [13]. In Khartoum hospital, Sudan-we documented that both maternal education and antenatal care inadequacy were associated with poor perinatal outcomes [14]. In neighboring Ethiopia, the level of education showed a significant association with the use of antenatal care [4]. Following this, we suggest that the low level of antenatal care use in this region could be countered with reproductive health education especially among multiparous women. Health education using a wide range of media should be a priority in order to increase awareness and use of antenatal care in this region. The study was conducted in eastern Sudan, where by tradition every pregnant and parous woman was married. One of the limitations of this study is that other factors like the social and economic status were not investigated because of practical difficulties. In Sudan, it is very difficult to investigate socio-economic status because of the extended families and the generous behaviors between the families and neighbors. The marital status and the social class were not to be associated with antenatal care inadequacy in the other setting [17]. If these were investigated in this study, our results might have been changed. The relationship between ANC and maternal outcomes is generally confounded by socio-demographic factors such as education and residence, however several studies have shown that low attendance of ANC is related to higher risk of maternal mortality [18,19].

In summary antenatal care showed a low coverage in Kassala, eastern Sudan. The inadequacy of antenatal care utilization was associated with high parity and low husband education. There should be more efforts to increase the coverage of antenatal care especially among women with high parity.

## Competing interests

The authors declare that they have no competing interests.

## Funding

The study was funded by University of Khartoum, Khartoum, Sudan.

## Authors' contributions

AAA and MMO carried out the study and participated in the statistical analysis and procedures. IA, AOA coordinated and participated in the design of the study, statistical analysis and the drafting of the manuscript. All the authors read and approved the final version.

## Pre-publication history

The pre-publication history for this paper can be accessed here:

http://www.biomedcentral.com/1471-2393/10/67/prepub
